# A High-Precision Method for Warehouse Material Level Monitoring Using Millimeter-Wave Radar and 3D Surface Reconstruction

**DOI:** 10.3390/s25092716

**Published:** 2025-04-25

**Authors:** Wenxin Zhang, Yi Gu

**Affiliations:** School of Automation, Beijing Information Science & Technology University, Beijing 100192, China; gy1360420733@outlook.com

**Keywords:** millimeter-wave radar, multi-feature fusion, point cloud processing, surface reconstruction, volume measurement

## Abstract

This study presents a high-precision warehouse material level monitoring method that integrates millimeter-wave radar with 3D surface reconstruction to address the limitations of LiDAR, which is highly susceptible to dust and haze interference in complex storage environments. The proposed method employs Chirp-Z Transform (CZT) super-resolution processing to enhance spectral resolution and measurement accuracy. To improve grain surface identification, an anomalous signal correction method based on angle–range feature fusion is introduced, mitigating errors caused by weak reflections and multipath effects. The point cloud data acquired by the radar undergo denoising, smoothing, and enhancement using statistical filtering, Moving Least Squares (MLS) smoothing, and bicubic spline interpolation to ensure data continuity and accuracy. A Poisson Surface Reconstruction algorithm is then applied to generate a continuous 3D model of the grain heap. The vector triple product method is used to estimate grain volume. Experimental results show a reconstruction volume error within 3%, demonstrating the method’s accuracy, robustness, and adaptability. The reconstructed surface accurately represents grain heap geometry, making this approach well suited for real-time warehouse monitoring and providing reliable support for material balance and intelligent storage management.

## 1. Introduction

Food security is a critical factor for economic and social stability. Key objectives include maintaining accurate inventory levels, preserving grain quality, and ensuring secure storage conditions [1]. However, managing large-scale grain reserves over extended storage periods presents significant challenges, particularly in supervision, transparency, and stock discrepancies. These issues can lead to inventory losses and fraudulent reporting.

To mitigate these risks, regulatory authorities perform regular grain stock inspections. These include a nationwide inventory census conducted once every decade and annual random audits assessing inventory levels, grain quality, and compliance with trade policies [2]. Despite these measures, current inspection methods still rely heavily on manual verification, which is inefficient, labor-intensive, and costly [3]. Consequently, achieving high-precision and real-time monitoring for modern grain storage management remains a challenge.

Traditional grain level detection methods are becoming obsolete, struggling to meet the demands for rapid and precise grain volume estimation. As a result, accurate stockpile assessment remains a critical challenge for warehouse operators and grain storage authorities [4]. Recent advancements in measurement and sensing technologies have facilitated the development of various inventory monitoring techniques, including pressure sensors [5] three-dimensional (3D) laser scanning [6], ultrasonic sensors, radar systems, and image-based analysis [7,8]. While these methods have improved measurement accuracy, they still exhibit significant limitations in complex warehouse environments. For instance, 3D laser scanning is highly susceptible to dust and obstructions, making it difficult to capture complete point cloud data in storage settings [9].

In contrast, millimeter-wave (MMW) radar, with its high resolution and strong penetration capabilities [10], can maintain stable and reliable measurements even in environments with dust, haze, and smoke. This makes it highly suitable for real-time monitoring in warehouses, mining sites, and other industrial applications [11]. MMW radar is an advanced electromagnetic sensing technology [12,13] that determines target position, distance, and velocity by transmitting electromagnetic waves and analyzing their echoes [14].

Among different radar configurations, frequency-modulated continuous-wave (FMCW) MMW radar is widely used in civilian and military applications due to its low complexity, high resolution, and strong stability [15]. In addition to providing high-precision measurements [16], FMCW MMW radar exhibits exceptional environmental adaptability, ensuring stable operation even in challenging conditions such as dust, haze, and low-visibility environments [17].

Despite these advantages, deploying MMW radar for warehouse monitoring presents several technical challenges. Multipath effects and weak reflection intensities in radar signal processing can cause anomalous signal jumps, reducing measurement accuracy. Additionally, MMW radar-acquired point cloud data are typically sparse, making it difficult to capture the complex geometric features of grain heaps accurately [18]. Addressing these challenges requires integrating data correction and optimization techniques to enhance monitoring precision and robustness [19].

Among existing 3D surface reconstruction algorithms, the Non-Uniform Rational B-Spline (NURBS) surface fitting method is widely used due to its computational efficiency and real-time processing capabilities [20]. However, NURBS-generated surfaces often appear overly smooth and lack the necessary details to accurately represent the intricate grain surface geometry.

To overcome these limitations, this study proposes a warehouse level monitoring method that integrates MMW radar with 3D surface reconstruction techniques. We introduce an anomalous signal correction method that fuses angle and range features. This approach analyzes the spatial distribution and energy characteristics of radar echoes to correct abnormal points and enhance data reliability. MMW radar is employed to acquire point cloud data of the grain surface, which are then preprocessed before undergoing 3D surface reconstruction [21] to generate a high-fidelity geometric model of the grain heap. This model is subsequently used to calculate the internal grain volume of the warehouse.

Three-dimensional surface reconstruction is a key technique in reverse engineering and is widely applied in industrial inspection and digital modeling. Depending on the reconstruction approach, 3D surface reconstruction can be classified into mesh-based, parametric, and implicit surface reconstruction. Mesh-based reconstruction typically relies on triangulation, which becomes computationally expensive when handling dense point clouds. Parametric surface reconstruction generates models via interpolation and fitting but is highly sensitive to noise and outliers, potentially leading to overfitting.

In contrast, implicit surface reconstruction techniques [22], such as Poisson Surface Reconstruction (PSR) [23], generate smooth and continuous surfaces by minimizing an objective function. This makes them well suited for reconstructing complex geometries, even in noisy and sparse data conditions [24]. Implicit reconstruction is particularly advantageous for capturing the irregular and natural accumulation patterns of grain heaps [25].

By integrating MMW radar with surface reconstruction techniques, the proposed method effectively addresses key challenges related to anomalous signal correction and sparse point cloud data, providing a high-precision, cost-effective solution for warehouse-level monitoring. Experimental results confirm that the proposed approach significantly improves monitoring accuracy and adaptability, supporting automated and intelligent grain warehouse management.

**Figure 1:**
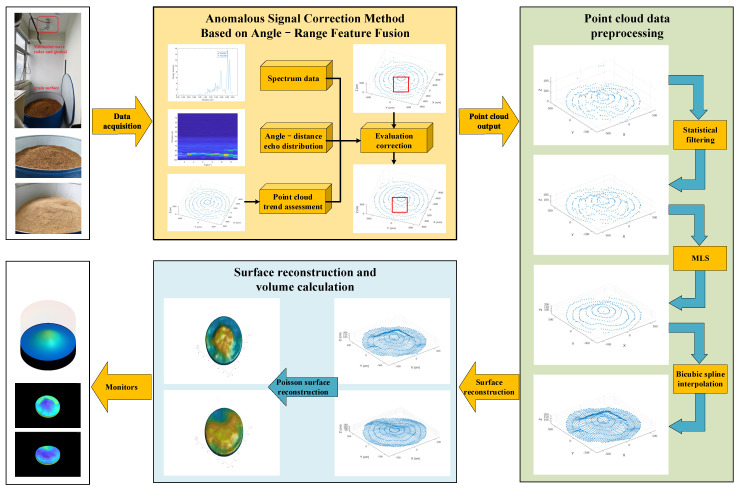
Overall flow diagram.

This study investigates the use of MMW radar to acquire warehouse grain surface point cloud data, as illustrated in Figure 1. We process these data using enhancement and surface reconstruction algorithms to generate a 3D geometric model of the grain surface. The ultimate goal is to achieve accurate grain volume estimation by computing the enclosed volume. The main contributions of this work are as follows:

- Utilization of FMCW MMW radar and Chirp-Z Transform (CZT) super-resolution algorithms to obtain high-precision warehouse point cloud data. A novel anomalous signal correction method is introduced to mitigate measurement errors from multipath effects and weak reflections, thereby improving data quality and accuracy.
- Implementation of preprocessing techniques, including denoising, smoothing, outlier removal, and data enhancement, to refine the acquired 3D point cloud data. These processes enhance data integrity and provide a robust foundation for accurate surface reconstruction.
- Application of Poisson Surface Reconstruction to generate a high-fidelity 3D geometric model from the processed point cloud data. We then apply the vector triple product method to calculate the enclosed volume, enabling precise grain volume estimation in storage facilities.

The remainder of this paper is organized as follows: Section 2 presents the principles and technical challenges of millimeter-wave radar in warehouse monitoring. Section 3 details the proposed data processing methods, including signal correction, statistical filtering, smoothing, and surface reconstruction. Section 4 describes the experimental setup, evaluates the proposed method through simulations and real-world experiments, and compares its performance with conventional approaches. Finally, Section 5 concludes this study and discusses potential directions for future research.

## 2. Application and Technical Challenges of Millimeter-Wave Radar

During grain loading and unloading in warehouses, significant amounts of dust and haze are often present, and interior lighting is typically insufficient. Under these challenging conditions, millimeter-wave (MMW) radar excels with its superior penetration and high-resolution capabilities, ensuring real-time and accurate warehouse monitoring. Specifically, MMW radar enables precise warehouse assessment, including stock levels, grain consumption rates, and material balance regulation. These functionalities provide reliable data support for warehouse management and operations.

This section introduces the principles and characteristics of MMW radar, explores the application of Chirp-Z Transform (CZT) super-resolution technology for improved measurement accuracy, and discusses methods for analyzing raw data to correct target point anomalies.

### 2.1. FMCW Millimeter-Wave Radar Distance Measurement Principle

Frequency-modulated continuous-wave (FMCW) millimeter-wave radar is a high-precision ranging technology based on linear frequency modulation (LFM) signals. It offers superior measurement resolution, robust penetration, and strong anti-interference capabilities, making it ideal for target detection and distance estimation in complex warehouse environments.

**Figure 2:**
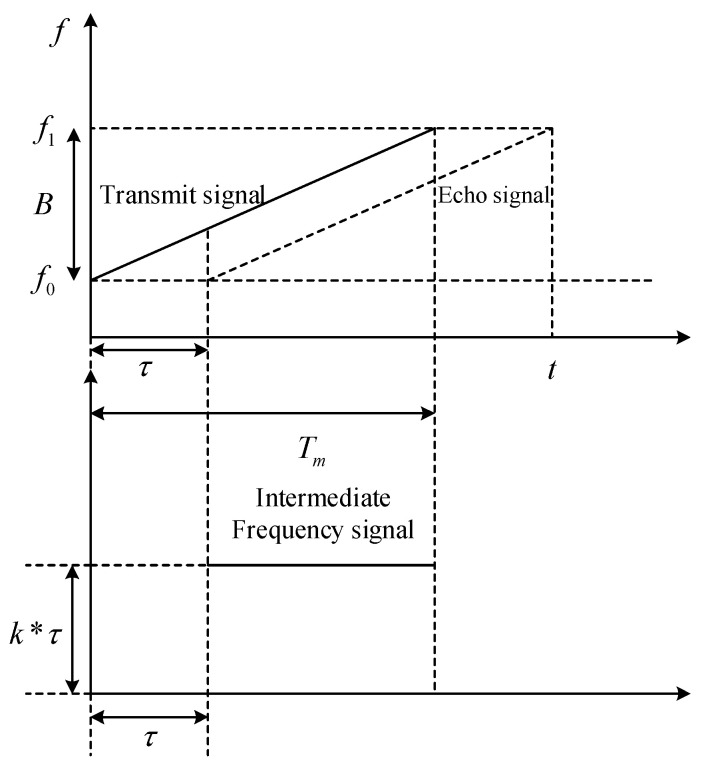
Time–frequency diagram of transmit signal, echo signal, and IF signal.

The core principle of FMCW radar is based on the time delay between the transmitted frequency-modulated signal and the received echo signal. Through signal mixing, the system extracts the intermediate frequency (IF) signal, which is subsequently used for target distance calculation. The relationship between the transmitted frequency, received echo frequency, and time delay is visualized in Figure 2.

In an FMCW radar system, the transmitted signal is a linearly frequency-modulated waveform, where its frequency varies linearly over time. The signal can be mathematically expressed as follows:

(1) $$S_{\mathrm{tx}}(t)=A_{0}\cos\left(2\pi f_{0}t+\pi kt^{2}\right)$$

where $f_{0}$ is the initial frequency, k=B/T is the chirp rate, B represents the signal bandwidth, and T denotes the modulation period. When the transmitted signal encounters a target, the echo signal is received at the radar after a time delay τ=2R/c, where R is the target distance and c is the speed of light. Thus, the received echo signal is given by

(2) $$S_{\mathrm{rx}}(t)=A^{\prime }\cos\left[2\pi f_{0}(t-\tau )+\pi k{\left(t-\tau \right)}^{2}\right]$$

The system generates an intermediate frequency (IF) signal by mixing the transmitted and received signals. Since the time delay τ is much smaller than the modulation period T, the IF signal frequency $f_{IF}$ can be approximated as follows:

(3) $$f_{IF}=\frac{2kR}{c}$$

The IF maintains a linear relationship with the target distance R. Therefore, performing frequency spectrum analysis using the Fast Fourier Transform (FFT) to extract $f_{IF}$ allows the target distance can be computed as follows:

(4) $$R=\frac{c\cdot f_{IF}}{2k}$$

In summary, FMCW millimeter-wave radar achieves high-precision distance measurement by transmitting linear frequency-modulated signals and processing the intermediate frequency of the received echoes. Its technical advantages include strong anti-interference capabilities and adaptability to complex environments, making it particularly well suited for grain warehouse level monitoring in dusty and hazy conditions [14].

### 2.2. CZT (Chirp-Z Transform) Super-Resolution

The Chirp-Z Transform (CZT) is a spectral refinement algorithm that enables high-resolution frequency analysis by selectively sampling regions in the complex plane. Unlike the conventional Fast Fourier Transform (FFT), which uniformly samples across the entire frequency domain, CZT enables localized spectral refinement, enhancing frequency resolution in specific target regions. This capability makes CZT particularly effective for target detection and high-precision signal processing.

By adjusting the starting point and transformation path, CZT enables flexible, high-resolution spectral magnification. In practical applications, the raw echo signal undergoes preprocessing to enhance signal quality and minimize distortions. This process involves removing DC components and applying a windowing function, such as a Hamming window, to reduce edge effects. Next, an FFT is performed to obtain an initial spectral analysis of the received echo signal, identifying the peak frequency of the reflected signal. CZT is then applied to the spectral peak region, achieving localized spectral magnification and high-precision frequency estimation.

**Figure 3:**
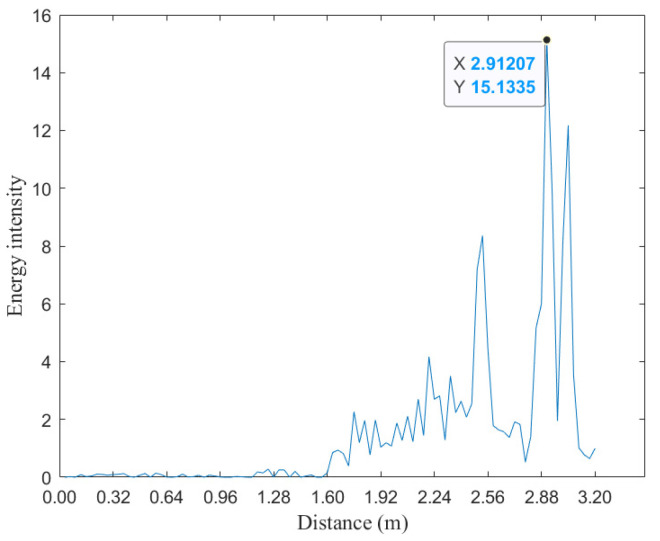
FFT spectrum analysis diagram.

**Figure 4:**
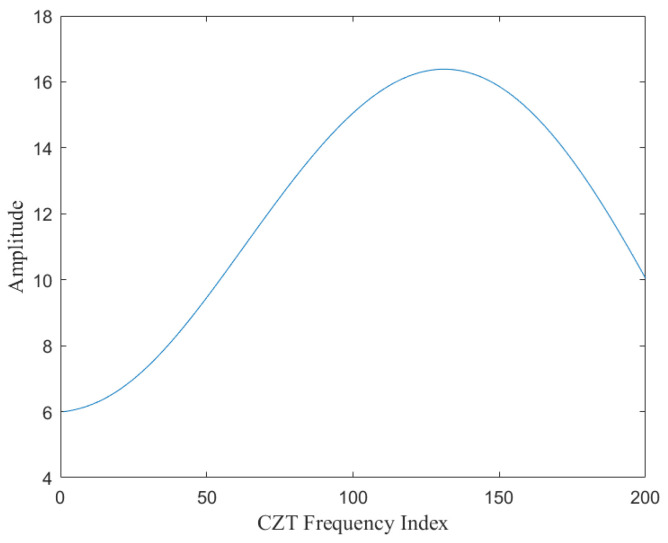
CZT spectrum analysis diagram.

As shown in Figure 3, a typical FFT spectrum analysis reveals multiple frequency peaks corresponding to the echo signal. Conventionally, the strongest peak is selected as the target point. However, in cases of weak reflections, overlapping echoes, or multiple targets, the FFT spectrum often lacks sufficient resolution for precise estimation. By applying CZT to the region of interest, as illustrated in Figure 4, the spectral resolution is significantly enhanced, improving both accuracy and clarity. This refined spectral analysis is particularly beneficial in scenarios involving weak target echoes, severe multipath interference, or multiple-object clutter. Additionally, CZT utilizes fast convolution-based computation, significantly reducing computational complexity while ensuring real-time signal processing. This efficiency makes CZT a highly effective tool for improving radar measurement accuracy in practical applications.

In conclusion, CZT super-resolution technology effectively overcomes the resolution limitations of traditional FFT by performing localized high-resolution spectral analysis in FMCW millimeter-wave radar signal processing. By significantly improving target distance estimation accuracy and robustness, CZT plays a crucial role in enabling precise and reliable millimeter-wave radar applications in complex environments.

### 2.3. Anomalous Signal Correction Method Based on Angle–Range Feature Fusion

In FMCW millimeter-wave radar systems, weak reflection intensity and multipath effects introduce interference into the raw echo signal, often resulting in multiple peaks in the signal spectrum. As a result, accurately identifying the true reflection point at the Field-of-View (FOV) center becomes challenging. To address this issue, we propose an anomalous signal correction method that fuses angle–range features, analyzing radar echo intensity, angular distribution, and overall trends in point cloud data. This approach enables multi-dimensional optimization and correction of raw measurements, significantly improving measurement accuracy.

We first perform FFT spectrum analysis to detect candidate peak positions; however, these results serve only as a reference. The final determination of the target point requires integrating angle–range distribution features and spatial continuity constraints to enhance accuracy. As illustrated in Figure 3, the radar echo spectrum often contains multiple peaks with varying reflection intensities. Conventional radar ranging methods typically select the strongest peak as the target point for CZT super-resolution processing. However, in cases where the FOV center reflection intensity is weak or affected by multipath interference, the actual reflection point may not correspond to the highest spectral peak. Consequently, relying solely on the strongest peak may lead to misjudgment, necessitating a more comprehensive analysis.

**Figure 5:**
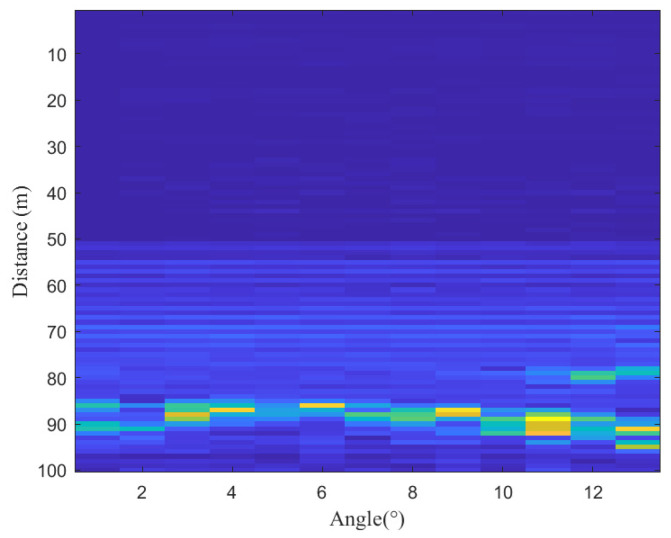
Angle–distance echo intensity distribution.

As shown in Figure 5, the relationship between angular distribution and energy intensity at radar measurement points reveals the overall distribution trend of the grain surface. In the figure, points with higher energy intensities (depicted in brighter colors) correspond to the spectral peaks in Figure 3. The continuity of the grain surface’s energy distribution across different angles provides a robust basis for correcting anomalous spectral peaks.

To improve target point determination accuracy, a multi-dimensional analysis is performed by integrating angle–range features. For each candidate target point $p_{i}$, its angle $\theta_{i}$ and radial distance $R_{i}$ are used, along with the echo intensity I(θ,R), to construct an angle–range feature space. The confidence score $S(p_{i})$ of each candidate target point is defined as a weighted combination of angle continuity $C_{a}(p_{i})$ and energy trend consistency $C_{e}(p_{i})$:

(5) $$S(p_{i})=\alpha \cdot C_{a}(p_{i})+\beta \cdot C_{e}(p_{i})$$

where α and β are weighting coefficients, satisfying α+β=1.

Angle continuity is measured by computing the variance of angular differences $\sigma_{\theta }^{2}$ within the local neighborhood of the target point, which reflects the smoothness of the angular distribution. A smaller variance indicates a smoother and more continuous angular distribution:

(6) $$C_{a}(p_{i})=\frac{1}{1+\sigma_{\theta }^{2}}$$

Energy trend consistency is evaluated by performing a linear fit on the energy intensities of neighboring points and calculating the fitting residuals to measure how well the energy trends match. The difference between the actual energy intensity $E_{j}$ at each neighboring point and the fitted value ${\hat{E}}_{j}$ is quantified using

(7) $$C_{e}(p_{i})=\frac{1}{1+\sum_{j=1}^{K}\left|E_{j}-{\hat{E}}_{j}\right|}$$

where K represents the number of neighboring points.

To further eliminate abnormal peaks, the overall spatial distribution trend of the point cloud is assessed. In typical grain warehouse environments, the grain surface exhibits a smooth and continuous spatial distribution. Therefore, local spatial characteristics can be used to detect and remove jump points and outliers. For each $P_{i}$, a local density consistency indicator $D(P_{i})$ is defined to measure the spatial consistency between $P_{i}$ and its neighboring points:

(8) $$D(P_{i})=\frac{1}{K}\sum_{j=1}^{K}\exp\left(-\frac{||P_{i}-P_{j}|{|}^{2}}{{2r_{n}}^{2}}\right)$$

where $r_{n}$ is the neighborhood radius, and K is the number of neighboring points; if $D(p_{i})$ falls below a threshold τ, the point $p_{i}$ is classified as an outlier or jump point. This process effectively eliminates anomalous points caused by multipath effects or weak reflections, ensuring that the selected target points are reliable and consistent.

Based on the angle–range feature fusion analysis and the overall trend evaluation of point cloud data, the anomalous signal correction algorithm follows the following steps:

1. Input: radar echo spectral data and point cloud data $P=\left\{p_{1},p_{2},\ldots ,p_{N}\right\}$.
2. Spectral Analysis: perform FFT analysis on the radar echo data, extract candidate peak values $\left\{f_{1},f_{2},\ldots ,f_{N}\right\}$, and select the strongest peak as the initial target point.
3. Angle–Range Feature Fusion: compute the confidence score $S(p_{i})$ for each candidate point $p_{i}$, evaluating angle continuity and energy trend consistency.
4. Point Cloud Trend Evaluation: use the local density consistency indicator $D(p_{i})$ to further detect and eliminate abnormal points.
5. Anomalous Signal Correction: filter out unreliable target points based on angle–range features and point cloud trend evaluation, retaining only the points with a high confidence score $S(p_{i})$ and density consistency $D(p_{i})$ above a threshold τ as the final corrected target position.

By applying this method, we effectively filter multiple peaks in raw radar data while integrating angle–range feature fusion for anomalous signal correction. This significantly improves target point determination accuracy and provides a high-precision foundation for subsequent point cloud processing and 3D surface reconstruction.

## 3. Point Cloud Data Processing and Surface Reconstruction

Millimeter-wave radar data are often affected by environmental factors like dust and complex objects, introducing numerous discrete outliers and noise points. These undesired points significantly impact surface reconstruction and volume estimation by causing triangular mesh vertices in the reconstructed surface to deviate from the actual model, leading to artificial surfaces (false faces).

To tackle these challenges, we propose an efficient data cleaning and filtering approach that removes noise while preserving valid data, ensuring accuracy in subsequent processing. After removing outliers, gaps may appear in the point cloud data, potentially compromising surface reconstruction precision. To mitigate this issue, we employ point cloud enhancement techniques, including interpolation and extrapolation, to fill missing data. This ensures a more complete and continuous representation before performing surface reconstruction and volume estimation.

### 3.1. Outlier Removal in Point Cloud Data

Point cloud data acquired via millimeter-wave radar are inherently affected by measurement noise, external interference, and sensing device precision limitations. Consequently, point cloud density distribution is often non-uniform, making it susceptible to outliers or noise points. These outliers exhibit irregular spatial distributions that disrupt the continuity and geometric integrity of the point cloud, degrading subsequent smoothing, surface reconstruction, and volume estimation.

To mitigate these effects, we employ a statistical filtering method based on Euclidean distance classification to detect and eliminate outliers. This method computes the average Euclidean distance of each point within its local neighborhood and classifies it as an outlier based on global statistical properties. Removing such outliers significantly improves the overall quality of the point cloud data.

The core steps of the statistical filtering approach are as follows:

- Local neighborhood determination: For each point $p_{i}$ in the point cloud data, the K-nearest neighbor (KNN) search method is applied to identify its K nearest neighbors, including the point itself. The selection of K is crucial as it reflects the local geometric characteristics in the point cloud. For dense point clouds, a larger K is used for dense point clouds to reduce local fluctuations, whereas a smaller K is preferable for sparse point clouds to preserve fine details.
- Computation of average neighborhood distance: After determining the K nearest neighbors for each point, the point itself is excluded, resulting in K−1 actual neighbors being used for local analysis. The average Euclidean distance between $p_{i}$ and its remaining *K* − 1 neighbors is then computed as follows:
  (9) $$d_{i}=\frac{1}{K-1}\sum_{j=1}^{K-1}∥p_{i}-p_{j}∥$$
  where $∥p_{i}-p_{j}∥$ represents the Euclidean distance between point $p_{i}$ and its neighboring point $p_{j}$.
- Global statistical feature computation and threshold setting: The global statistical properties of the point cloud are computed using the mean μ and standard deviation $\sigma_{d}$ of the average neighborhood distances:
  (10) $$\mu =\frac{1}{N}\sum_{i=1}^{N}d_{i},\;\sigma_{d}=\sqrt{\frac{1}{N}\sum_{i=1}^{N}{\left(d_{i}-\mu \right)}^{2}}$$
  where N represents the total number of points in the point cloud. An outlier threshold T is defined as
  (11) T=μ+λ⋅σ
  where λ is the threshold coefficient, which controls filtering strictness.
- Outlier detection and removal: Each point is classified based on its average neighborhood distance $d_{i}$. If $d_{i}>T$, the point is classified as an outlier and removed; otherwise, it is retained. The final output is the filtered point cloud dataset.

**Figure 6:**
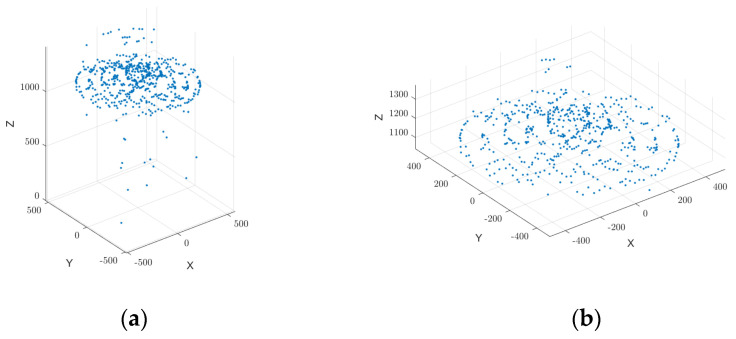
Comparison of the effect of applying statistical filtering to raw data. (**a**) Raw point cloud data. (**b**) Applying statistical filtering to point cloud to filter discrete points.

In summary, the statistical filtering method based on Euclidean distance classification effectively eliminates outliers and noise points from millimeter-wave radar point cloud data, as illustrated in Figure 6. By incorporating local neighborhood analysis and global threshold determination, this method enhances the accuracy and reliability of the point cloud data. The refined data provide a high-quality foundation for subsequent 3D surface reconstruction and volume estimation.

### 3.2. Point Cloud Smoothing

Removing discrete outliers from millimeter-wave radar-scanned point cloud data improves overall quality. However, local edge protrusions may still cause irregularities, affecting dataset smoothness. These spikes and discontinuities disrupt surface smoothness and may form sharp anomalies during reconstruction, reducing model accuracy and continuity. To further refine the geometric characteristics of the point cloud data, we apply the Moving Least Squares (MLS) method for smoothing.

The Moving Least Squares (MLS) method is a widely adopted weighted fitting technique designed to smooth point cloud data by approximating a low-order polynomial surface within a localized neighborhood. This is achieved through a weighted least squares approach, which effectively mitigates local fluctuations and enhances the continuity of the surface representation. For any given point $p_{i}$, the MLS smoothing process consists of the following steps:

- Neighborhood Determination: For each point $p_{i}$, the K-nearest neighbor (KNN) search method is used to determine its K closest neighbors, forming the local neighborhood. The size of K is a crucial parameter: a larger K increases smoothing effectiveness but may cause excessive blurring of fine geometric details, whereas a smaller K preserves sharp features but weakens the smoothing effect.
- Weight Function Construction: To ensure that neighboring points contribute appropriately to the local surface fitting, a Gaussian weight function is applied, prioritizing closer points while reducing the influence of distant neighbors. The weight function is defined as
  (12) $$w_{j}=\exp\left(-\frac{d_{j}^{2}}{{\sigma_{w}}^{2}}\right),\;j=1,2,\ldots ,K$$
  where $d_{j}$ represents the Euclidean distance between the point $p_{i}$ and its j nearest neighbor, and $\sigma_{w}$ is a weighting function parameter, typically set as the maximum distance within the neighborhood, ensuring that weights decay with increasing distance.
- Local Weighted Least Squares Fitting: After determining the neighborhood points and their weights, a local polynomial surface is fitted. A second-order polynomial model is adopted to describe the local surface [26,27]:
  (13) $$z=a_{0}+a_{1}x+a_{2}y+a_{3}x^{2}+a_{4}xy+a_{5}y^{2}$$
  where $a_{0}\sim a_{5}$ are the coefficients to be determined. These coefficients are obtained by constructing a weighted least squares error function:
  (14) $$E=\sum_{j=1}^{K}w_{j}{\left(a_{0}+a_{1}x_{j}+a_{2}y_{j}+a_{3}x_{j}^{2}+a_{4}x_{j}y_{j}+a_{5}y_{j}^{2}-z_{j}\right)}^{2}$$

By minimizing the error function E, the polynomial coefficients $a_{0}\sim a_{5}$ are computed, and the height value $z_{fitted}$ of the center point $p_{i}$ on the fitted surface is determined.

**Figure 7:**
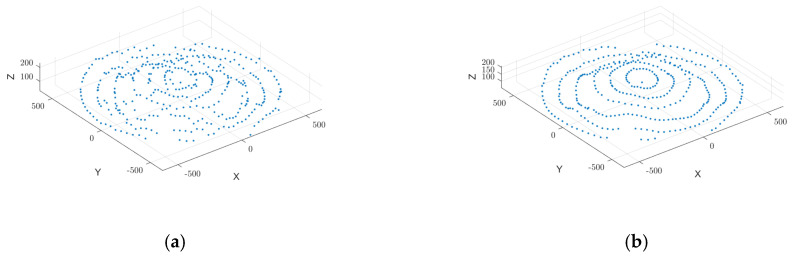
Applying MLS smoothing to data after statistical filtering. (**a**) Statistically filtered data; (**b**) MLS smoothing point cloud data.

The Moving Least Squares (MLS) smoothing technique effectively eliminates sharp surface irregularities and local spikes in millimeter-wave radar-acquired point cloud data, as illustrated in Figure 7. By fitting localized polynomials, MLS enhances dataset smoothness and structural integrity, ensuring a continuous surface representation. This method significantly improves the quality of input data for 3D surface reconstruction and volume estimation, leading to more accurate and stable measurement results.

### 3.3. Point Cloud Data Enhancement

In point cloud data preprocessing, interpolation methods play a critical role in improving resolution and quality. Appropriate interpolation techniques reconstruct missing points, enhancing data continuity and completeness. This ensures high-quality input for subsequent surface reconstruction.

In millimeter-wave radar-acquired point cloud data, discrete point removal and noise filtering often introduce local voids or incomplete regions. These gaps cause errors and discontinuities in surface reconstruction, reducing accuracy. To address this issue, we apply bicubic spline interpolation to enhance point cloud data. This method leverages locally continuous cubic polynomial functions, enabling precise data fitting. It effectively fills missing data regions while maintaining smoothness and continuity, ensuring reliable data for further processing.

Bicubic spline interpolation [28] is a high-order technique applicable to both 2D and 3D data. The fundamental principle involves applying cubic spline interpolation along each dimension of the point cloud to ensure continuity of the function and its first- and second-order derivatives across segments. The interpolation function is formulated as follows:

(15) $$S(x,y)=\sum_{s=0}^{n+2}\sum_{t=0}^{m+2}\alpha_{s}t\varphi_{s}(x)\psi_{t}(y)$$

where $\alpha_{s}t$ represents the interpolation coefficients to be determined, while $\varphi_{s}(x)$ and $\psi_{t}(y)$ are univariate cubic spline basis functions. The basis spline functions are defined as follows:

(16) $$y\left(x\right)=\left\{\begin{array}{ll} y\left(-x\right) & x<0\\ (3\lambda +2)x^{3}-3\left(\lambda +1\right)x^{2}+1 & 0⩽x<1\\ 3\lambda^{j}\left[(\lambda +1){\left(x-j\right)}^{3}-\left(\lambda +2\right){\left(x-j\right)}^{2}+\left(x-j\right)\right] & j⩽x<j+1 \end{array}\right.$$

where $\lambda =\sqrt{3}-2$ is a fixed parameter that ensures continuity of the first and second derivatives at x=0 and x=1. By using sampling point coordinate values, the input space is divided into a grid of n×m sub-rectangular grids. The bicubic spline function computes sampling points along the x and y axes, utilizing the corresponding cubic spline basis functions $\varphi_{s}(x)$ and $\psi_{t}(y)$. These basis functions are then combined with boundary node first-order partial derivatives and vertex second-order partial derivatives to solve for interpolation coefficients $\alpha_{s}t$. Finally, given interpolation points $\left(Ix_{i},Iy_{i}\right)$, the interpolation result is obtained by solving $S(Ix_{i},Iy_{i})$.

**Figure 8:**
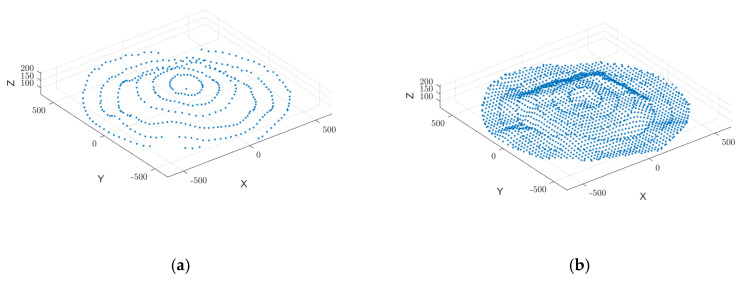
Point cloud data enhancement. (**a**) MLS smoothing point cloud data. (**b**) Strengthening point cloud data by bicubic spline interpolation.

By applying bicubic spline interpolation, missing data regions are reconstructed, filling voids and generating new sampling points, as illustrated in Figure 8. This process significantly improves the smoothness and continuity of the point cloud, ultimately establishing a robust foundation for 3D surface reconstruction and volume estimation.

### 3.4. Poisson Surface Reconstruction

Poisson Surface Reconstruction (PSR) is a widely recognized technique for robustly handling noisy and discrete point cloud data while ensuring surface smoothness [22]. The core principle of this method is to reformulate the surface reconstruction problem as a Poisson equation, integrating and optimizing the divergence of the normal vector field in point cloud data. This approach derives an implicit surface equation, facilitating high-quality mesh extraction [23,24].

**Figure 9:**
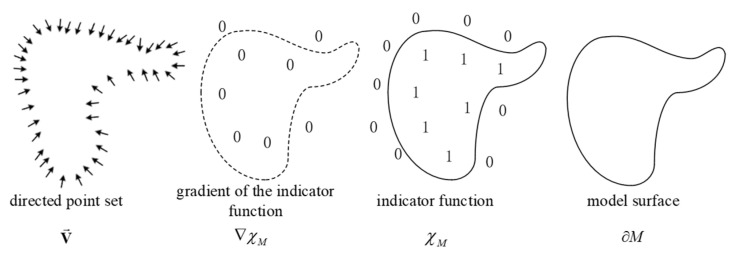
Poisson reconstruction process.

PSR fundamentally relies on point cloud normal vectors with consistent orientations. Given a set of point cloud data $\left\{p_{i}\right\}$ and their corresponding normal vectors $\left\{n_{i}\right\}$, the method constructs a continuous vector field V(x) that converts discrete normal vector distributions into a field representation. This vector field is typically defined as the gradient of an indicator function χ(x), where χ(x)=1 inside the object and gradually tends to 0 outside, ensuring that the reconstructed surface aligns with an isosurface of the function. In an ideal case, illustrated in Figure 9, if χ(x) is a smooth indicator function where the inside of the object is labeled as 1 and the outside as 0, then its gradient ∇χ(x) will exhibit consistent alignment with surface normal vectors near the object boundary. By sampling the input normal vectors $\left\{n_{i}\right\}$ into a global vector field V(x), the reconstruction problem can be formulated as an optimization problem based on the Poisson equation. Mathematically, this requires finding a function χ(x) that satisfies the following condition:

(17) $$\nabla^{2}\chi (x)=\nabla \cdot V(x)$$

where $\nabla^{2}$ is the Laplacian operator, quantifying the curvature and variation in the scalar field χ(x,y,z) in 3D Euclidean space:

(18) $$\nabla^{2}\chi =\frac{\partial^{2}\chi }{\partial x^{2}}+\frac{\partial^{2}\chi }{\partial y^{2}}+\frac{\partial^{2}\chi }{\partial z^{2}}$$

The divergence of the vector field ∇⋅V is defined as follows:

(19) $$\nabla \cdot V=\frac{\partial V_{x}}{\partial x}+\frac{\partial V_{y}}{\partial y}+\frac{\partial V_{z}}{\partial z}$$

By solving the Poisson equation, the problem of surface reconstruction is transformed into a global optimization problem.

In practical applications, an Octree data structure is often used to hierarchically partition space, making it suitable for handling irregular and sparsely distributed point cloud data. By constructing basis functions at each Octree node, normal vector information and point distributions are aggregated and weighted, leading to the discretization of χ(x) and V(x) within the Octree framework. After discretization, the Poisson equation is transformed into a system of linear equations, which is then solved using numerical methods such as the Finite Element Method (FEM) or the Finite Difference Method (FDM). Once the implicit function χ(x) is obtained in discrete form, the Marching Cubes algorithm extracts the final 3D surface mesh. This ensures that the reconstructed surface maintains global consistency and smoothness, effectively reducing noise while preserving the overall geometric structure.

Poisson Surface Reconstruction globally integrates sampled points and normal vectors to generate an optimal implicit surface model [25]. This method significantly enhances the smoothness and consistency of reconstructed surfaces, making it well suited for modeling the complex natural pile shapes of grain storage environments. By providing a high-precision and high-quality geometric model, PSR supports accurate volume estimation and further computational analysis.

## 4. Experimental Results and Analysis

### 4.1. Experimental Setup

**Figure 10:**
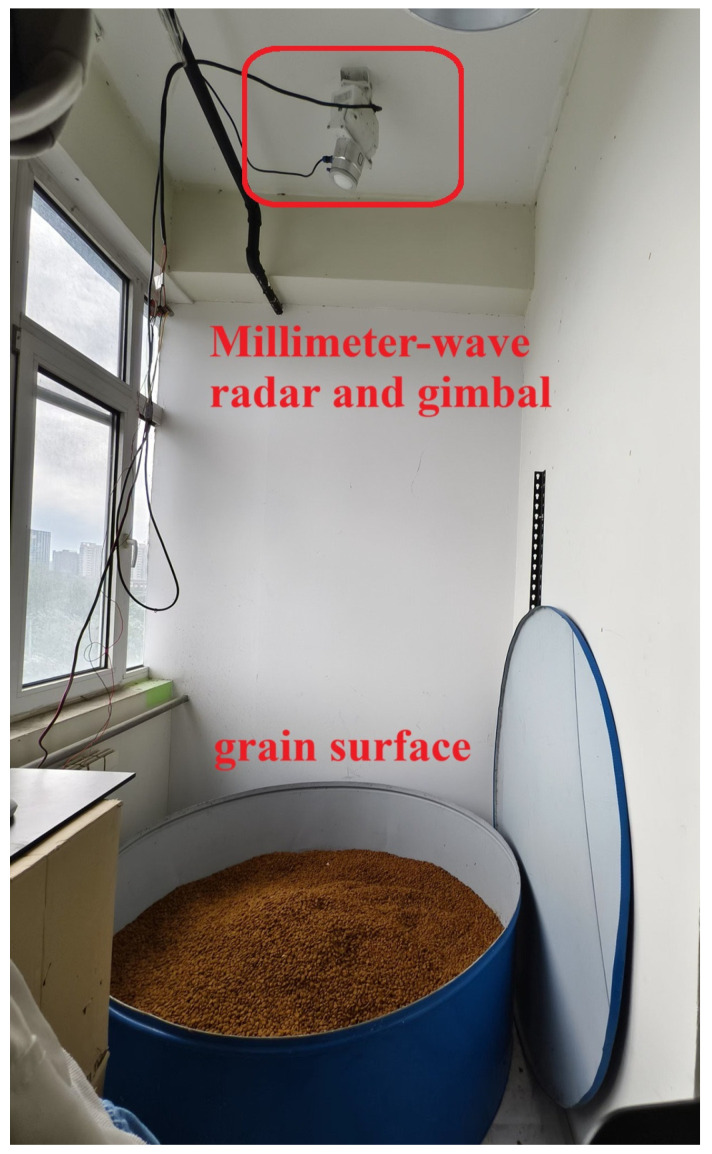
Three-dimensional level meter device based on millimeter-wave radar.

Data collection was performed in a simulated small-scale grain storage facility. The storage container was a cylindrical tank with a 65 cm radius, and after manually leveling the material, the height reached 11.5 cm, resulting in a measured volume of 152,564.75 cm^3^. The millimeter-wave radar was positioned directly above the grain storage tank, with its orientation and scanning controlled by a gimbal system. For this application, the radar frequency was set to 1.6 GHz. In its idle state, pointing vertically downward, it was positioned 3.12 m above the ground (Figure 10).

### 4.2. Experimental Procedure and Analysis

**Figure 11:**
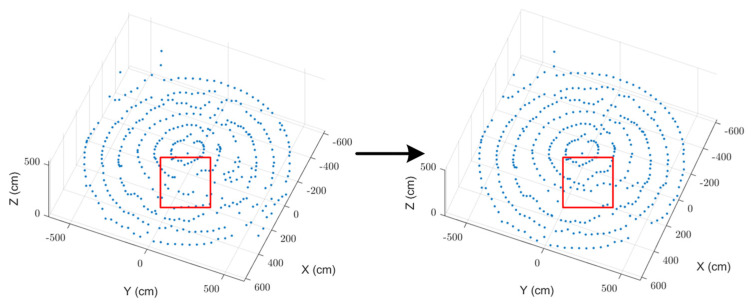
Singular point correction.

Millimeter-wave radar data showed anomalies like signal jumps due to weak reflections and multipath effects, resulting in inaccurate point cloud measurements. To address these issues, an angle–range feature fusion-based anomalous signal correction method was applied. By analyzing the angle–distance echo intensity distribution (Figure 5) and evaluating the overall point cloud trend, interfering peaks were removed, allowing for accurate localization of the grain surface. The corrected point cloud data (Figure 11) better aligned with the actual grain surface distribution and effectively removed outliers caused by interference. This correction improved data authenticity and ensured consistency.

Following the correction of anomalous points, statistical filtering based on Euclidean distance classification was applied to refine the point cloud. The parameters K (number of nearest neighbors) and λ (threshold coefficient) played a crucial role in controlling the filtering effect. A larger K meant more points were considered in the local neighborhood, increasing the constraints on the point cloud and removing more points. Conversely, a smaller λ imposed stricter filtering conditions, resulting in a reduction in retained point cloud data. For the initial dataset containing 393 points, the filtered results are shown in Table 1.

From Table 1, an appropriate selection of K and λ effectively removed outliers and noise while preserving key structural points of the grain surface.

**Figure 12:**
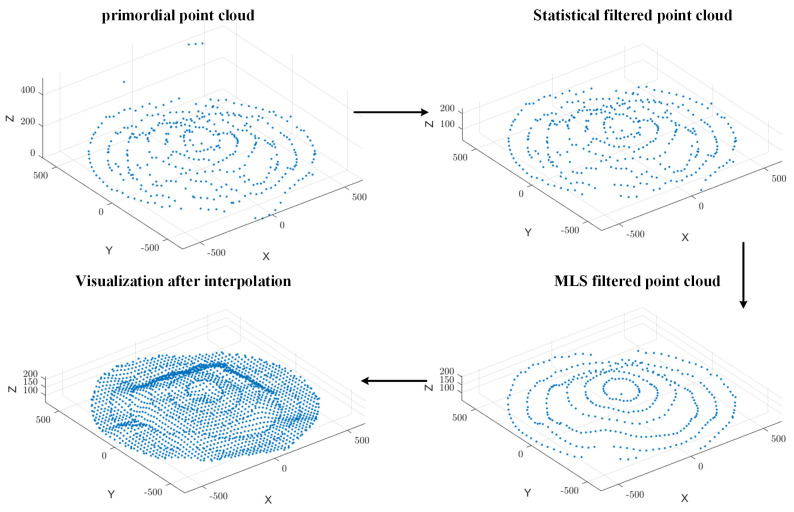
Point cloud data processing.

As illustrated in Figure 12, Moving Least Squares (MLS) smoothing was applied to refine the grain surface point cloud, removing spikes and discontinuities while improving uniformity. Bicubic spline interpolation further refined the dataset, filling voids and enhancing smoothness, establishing a solid foundation for 3D surface reconstruction and volume estimation.

**Figure 13:**
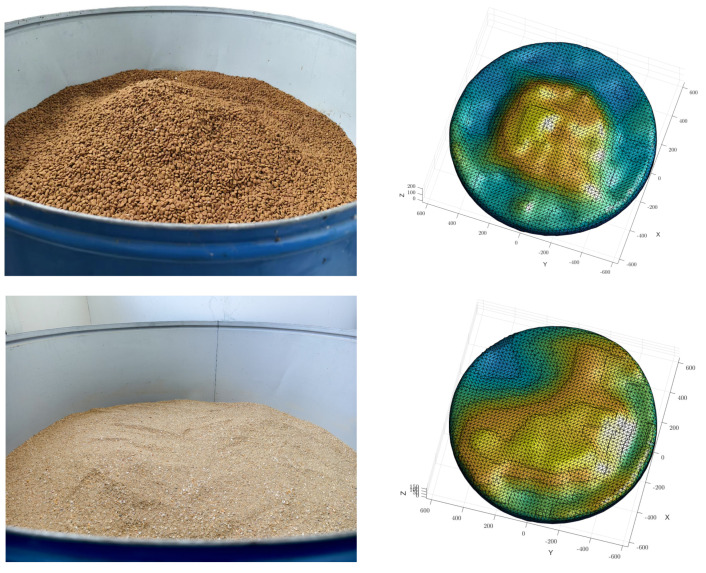
Comparison of different materials and shapes.

**Figure 14:**
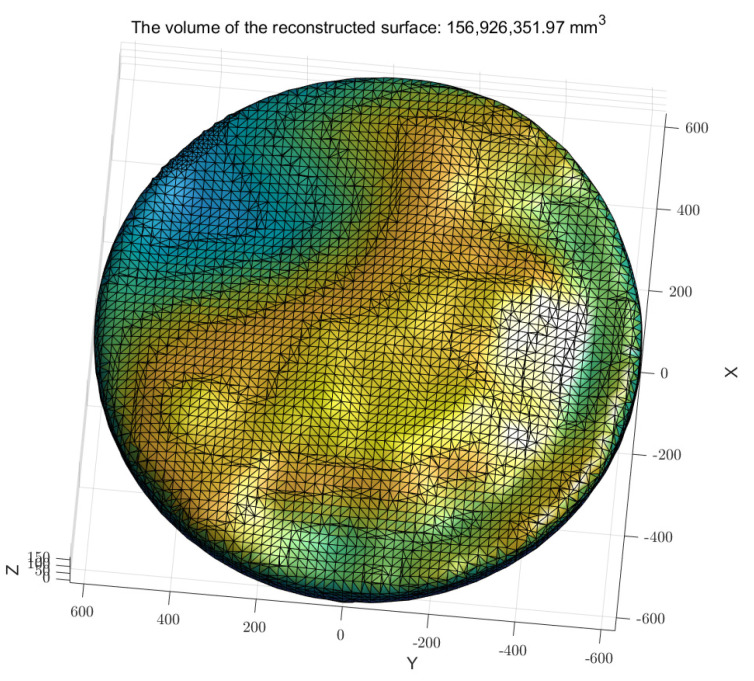
Volume calculation based on the reconstructed surface model.

After completing point cloud data cleaning and enhancement, a 3D surface model was generated using Poisson Surface Reconstruction (PSR). This method constructs an implicit function and optimizes both point cloud data and their normal vector field, producing a smooth and closed 3D surface, as shown in Figure 13. Once the reconstructed surface model was obtained, the vector triple product method was used to calculate its volume. Figure 14 illustrates the reconstructed model used for this calculation. As shown in Table 2, compared with the actual volume of 152,564.8 cm^3^, the relative error was less than 3%, demonstrating the accuracy and reliability of the reconstruction process.

## 5. Conclusions

This study presents a millimeter-wave radar-based method for grain warehouse level monitoring, integrating 3D reconstruction techniques to overcome the limitations of laser-based methods, which are affected by dust and fog in storage environments. The high penetration of millimeter-wave radar, combined with CZT super-resolution, enabled accurate grain surface measurements in complex environments. In the data processing stage, an angle–range feature fusion-based signal correction method eliminated interfering points, while statistical filtering, MLS smoothing, and bicubic spline interpolation significantly enhanced point cloud data quality. Finally, the Poisson Surface Reconstruction algorithm generated a high-precision 3D model. Experimental results showed a volume estimation error within 3%, validating the accuracy and robustness of the proposed method.

In conclusion, the proposed method demonstrates strong adaptability and reliability, offering an efficient and precise solution for grain storage monitoring and management.

## Figures and Tables

**Table 1:** Effect of different parameters on statistical filtering.

K	λ=0.1	λ=0.3	λ=0.5	λ=0.5
10	281	356	371	373
15	283	327	361	370
20	287	295	329	361

**Table 2:** Test of grain surface with different shapes.

	Measured Volume	Poisson Surface Reconstruction Volume	Relative Error
Shape 1	152,564.8 cm^3^	154,408.6 cm^3^	1.21%
Shape 2	152,564.8 cm^3^	156,926.4 cm^3^	2.86%

## Data Availability

https://pan.baidu.com/s/14d4vY6hj-LX1NwUXeGyRPw?pwd=q2d8 (accessed on 18 March 2025).
